# Enhanced osteogenic differentiation of alendronate-conjugated nanodiamonds for potential osteoporosis treatment

**DOI:** 10.1186/s40824-021-00231-9

**Published:** 2021-09-23

**Authors:** Guk Young Ahn, Sung-Eun Kim, Tae Hoon Yun, Inseong Choi, Daewon Park, Sung-Wook Choi

**Affiliations:** 1 Biomedical and Chemical Engineering, Department of Biotechnology, The Catholic University of Korea, 43 Jibong-ro Wonmi-gu, Gyeonggi-do 14662 Bucheon-si, Republic of Korea; 2 Department of Orthopaedic Surgery and Rare Diseases Institute, Korea University Medical Centre, Guro Hospital, 80 Guro-dong, Guro-gu, Seoul, 152-703 Republic of Korea; 3 Department of Bioengineering, University of Colorado Denver, Anschutz Medical Campus, 12800 E. 19th Avenue, Aurora, CO 80045 USA

**Keywords:** Alendronate, Nanodiamond, Proliferation, Osteogenic differentiation

## Abstract

**Background:**

Alendronate (Alen) is promising material used for bone-targeted drug delivery due to its high bone affinity and therapeutic effects on bone diseases. In addition, Alen can enhance the osteogenic differentiation of osteoblastic cell. Recently, nanodiamonds (NDs) with hardness, non-toxicity, and excellent biocompatibility are employed as promising materials for carrier systems and osteogenic differentiation. Therefore, we prepared Alen-conjugated NDs (Alen-NDs) and evaluated their osteogenic differentiation performances.

**Methods:**

Alen-NDs were synthesized using DMTMM as a coupling reagent. Morphological change of Mouse calvaria-derived preosteoblast (MC3T3-E1) treated with Alen-NDs was observed using the confocal microscope. The osteogenic differentiation was confirmed by cell proliferation, alkaline phosphatase (ALP), calcium deposition, and real-time polymerase chain reaction assay.

**Results:**

Alen-NDs were prepared to evaluate their effect on the proliferation and differentiation of osteoblastic MC3T3-E1 cells. The Alen-NDs had a size of about 100 nm, and no cytotoxicity at less than 100 μg/mL of concentration. The treatment of NDs and Alen-NDs reduced the proliferation rate of MC3T3-E1 cells without cell death. Confocal microscopy images confirmed that the treatment of NDs and Alen-NDs changed the cellular morphology from a fibroblastic shape to a cuboidal shape. Flow cytometry, alkaline phosphatase (ALP) activity, calcium deposition, and real-time polymerase chain reaction (RT-PCR) confirmed the higher differentiation of MC3T3-E1 cells treated by Alen-NDs, compared to the groups treated by osteogenic medium and NDs. The higher concentration of Alen-ND treated in MC3T3-E1 resulted in a higher differentiation level.

**Conclusions:**

Alen-NDs can be used as potential therapeutic agents for osteoporosis treatment by inducing osteogenic differentiation.

## Background

Osteoporosis is a metabolic disorder that increases the risk of fractures due to decreased bone density and strength, and deterioration of bone microstructure [1, 2]. It is difficult to know until a fracture occurs, leading to serious secondary problems of health, and even death [3, 4]. Various agents, including hormones (estrogen, calcitonin, and teriparatide) [5–7], selective estrogen receptor modulators (raloxifene and bazedoxifene) [8, 9], and bisphosphonates (alendronate, ibandronate, and zoledronate) [10, 11], have been studied for the treatment of osteoporosis. Among these agents, alendronate (Alen) is one of the bisphosphonates that have selective bone adsorption properties, preventing bone resorption by inhibiting osteoclastic activity, and one that is widely used in the treatment of osteoporosis [12, 13]. In addition, Alen is known to enhance the osteogenic differentiation of bone marrow stromal and osteoblastic cells [14, 15]. The currently available osteoporosis treatments are mostly conducted by oral tablets [16]. However, side effects, such as atypical fractures, atrial fibrillation, and osteonecrosis of the jaw, have occurred in oral drug delivery [17, 18]. Therefore, to prevent potential side effects, a suitable carrier system for delivering osteoporosis drugs is needed.

Recently, nanocarriers based on liposomes, polymers, silica, metal, hydroxyapatite, and carbon-based nanomaterials have been found to be promising for the effective treatment of osteoporosis [19]. Those nanocarriers have advantages in terms of protection from transport efficiency, biodegradation, targeting, biodistribution, and so forth [20]. Among these nanomaterials, nanodiamonds (NDs) with a truncated octahedral structure have emerged as promising carbon-based nanocarriers in a variety of biomedical applications, because of their optical properties, high surface area, non-toxicity, colloidal stability, good mechanical strength, and high surface functionality [21]. Zhang et al. fabricated ND-composited poly(l-lactic acid) scaffolds, and demonstrated their enhanced proliferation and differentiation on osteoblast through mRNA expression, suggesting the positive effect of NDs on osteoblastic differentiation [22]. Choi et al. designed Alen-modified hydroxyapatite nanoparticles and proved the capabilities of high affinities to tricalcium phosphate disk, bone-specific proliferation, and enhanced differentiation of osteoblast. The dual delivery effect of therapeutic Alen and osteoconductive hydroxyapatite showed great potential for the treatment of bone diseases [23].

We had demonstrated the bone-targeted ability of Alen-conjugated NDs (Alen-NDs) through in vivo study [24]. However, the effect of the Alen-ND on the cellular morphology and the feasibility for new bone formation were not evaluated in our previous work. Yi et al. investigated the cellular effects of gold nanoparticles (AuNPs) on the differentiation of mesenchymal stem cells (MSCs) and showed that the AuNPs promoted the osteogenic differentiation to osteoblast and inhibit adipogenic differentiation due to the mechanical stress on the cells [25]. In this work, we investigated the osteogenic differentiation effect of NDs as a functional carbon-based nanomaterial. Both the ND and Alen-ND changed the morphology of preosteoblasts and induced the transition from proliferation to differentiation, resulting in the secretion of bone-related biomarkers. To our best knowledge, it was the first report on the cellular morphological change and osteogenic differentiation of preosteoblasts by ND-based nanoparticles. We believe that Alen-NDs with the capability of inducing effective osteogenic differentiation could be a promising candidate for osteoporosis treatment.

## Materials and methods

### Materials

Carboxyl NDs and 4-(4,6-dimethoxy-1,3,5-triazine-2-yl)-4-methylmorpholinium chloride (DMTMM) were purchased from Tokyo Chemical Industry Co., Ltd. (TCI, Tokyo, Japan). Alendronate (Alen) was provided by Samjin Pharm. Co. Ltd. (Seoul, Korea). Dulbecco’s Modified Eagle’s Medium (DMEM), phosphate-buffered saline (PBS), and fetal bovine serum (FBS) were purchased from Welgene (Gyeongsan, Korea). Formaldehyde solution (4%) was purchased from T&I (Chuncheon, Korea). Cell Counting Kit-8 (CCK-8) was purchased from Dojindo Laboratories Co. Ltd. (Kumamoto, Japan).

### Synthesis of Alen-NDs

Alen-NDs were synthesized by forming an amide bond using DMTMM as a coupling reagent. ND powders (200 mg) were dispersed in 50 mL of deionized water. For conjugation of Alen onto the surface of NDs, DMTMM (10 mg) and Alen (100 mg) were added into the aqueous NDs dispersion, and stirred for 24 h at 60 °C, followed by centrifugation for purification with distilled water three times. The sample was then finally freeze-dried. To characterize ND-based nanoparticles, the dynamic light scattering method (Zeta-sizer Nano ZS, Malvern Instruments Ltd., Malvern, UK) was used to measure their sizes and zeta-potentials. Scanning electron microscopy (SEM, S-4800, Hitachi, Tokyo, Japan) was used to analyze their morphologies.

### Cell viability and proliferation

The cell viability of mouse fibroblast (NIH/3 T3) and mouse calvaria-derived preosteoblast (MC3T3-E1) cells were evaluated after the treatment of NDs and Alen-NDs. Aqueous dispersions of NDs (100 μg/mL) and Alen-NDs (10, 50, and 100 μg/mL) were added to each well containing 2 × 10^4^ cells/mL in a 96-well plate and incubated in a humidified atmosphere containing 5% CO_2_ at 37 °C. The culture medium consisted of DMEM supplemented with heat-inactivated 10% fetal bovine serum (FBS) and 1% antibiotics (penicillin and streptomycin). The number of cells was evaluated using the CCK-8 assay with respect to time. The CCK-8 solution (20 μL) was added to each well of the 96-well plates and maintained in an incubator for 1 h [26]. The sample extracts were transferred to 96-well plates, and their absorbances at 450 nm were measured by a microplate reader (Spectra Max Plus 384, Molecular Devices, Co. Ltd., Philadelphia, USA) [27]. The cell viability (%) was determined as the percentage of the absorbance of the treated group divided by the absorbance of the non-treated group (control) under the same condition.

Cell proliferation was measured using the CCK-8 assay at (1, 3, 5, and 7) days after cell seeding (2 × 10^4^ cells/mL). Aqueous dispersions of NDs (100 μg/mL) and Alen-NDs (only DMEM, 10, 50, and 100 μg/mL) were added to each well and cell proliferation was analyzed by the same method as in cell viability analysis.

### Cell morphology and differentiation

To confirm the morphological change of MC3T3-E1 cells, the cells (2 × 10^4^ cells/mL) were cultured in an 8-well Cell Culture Slide (SPL Life Sciences, Pocheon, Korea) containing 100 μL of the culture medium in an incubator. Aqueous dispersions of the only DMEM (control), NDs, and Alen-NDs (100 μg/mL) were added to each well and allowed to grow for 7 days. The culture slides were gently washed with PBS, fixed with formaldehyde solution (4%) for 15 min at room temperature, and rinsed 3 times with PBS. The cells were then stained with 4′,6-diamidino-2-phenylindole (DAPI) for 10 min and stained with rhodamine for 1 h in dark. The fluorescence was visualized by confocal microscopy (LSM710, Carl Zeiss, Oberkochen, Germany), and the cellular dimensions were analyzed using ImageJ® software (National Institutes of Health, Bethesda, USA).

The osteogenic differentiation was confirmed by fluorescence-activated cell sorting analysis (FACS, FACS Canto II, BD Biosciences, San Jose, USA). MC3T3-E1 cells (1 × 10^5^ cells/mL) were cultured after treatment by NDs and Alen-NDs (100 μg/mL) in the medium for 7 days. Then, anti-CD44, anti-CD51, and anti-CD45 antibodies (eBioscience) were treated to each well for 30 min on ice. After staining, cells were fixed with paraformaldehyde (2%) and analyzed with a FACS. Data were analyzed with FlowJo software (v. 10.1, FlowJo LLC, USA).

### ALP activity

To evaluate early osteogenic differentiation, MC3T3-E1 cells were seeded on a 24-well culture plate at a concentration of 1 × 10^5^ cells/mL, and incubated in culture medium (C.M., DMEM supplemented with 10% FBS and 1% antibiotics), osteogenic medium (O.M., C.M. supplemented with 50 μg/mL ascorbic acid, 10 nM dexamethasone, and 10 mM 훽-glycerophosphate), and culture medium containing NDs (100 μg/mL) or Alen-NDs (25, 50, and 100 μg/mL). The cells were obtained at predetermined time points of 3, 7, and 10 days. The cells were lysed using 1× RIPA (radioimmunoprecipitation assay) buffer [50 mM Tris–HCl, pH 7.4, 150 mM NaCl, 0.25% deoxycholic acid, 1% Tergitol-type-40 (NP-40), and 1 mM ethylenediaminetetraacetic acid (EDTA) including protease and phosphatase inhibitors (1 mM phenylmethylsulfonyl fluoride (PMSF), 1 mM sodium orthovanadate, 1 mM sodium fluoride, 1 μg/mL aprotinin, 1 μg/mL leupeptin, and 1 μg/mL pepstatin)]. The cell lysates were centrifuged at 13,500 rpm for 3 min at 4 °C. The supernatants were incubated with *p*-nitrophenyl phosphate solution for 30 min at 37 °C. The reaction was stopped by the addition of 500 μL of 1 N NaOH. ALP activity was determined by measuring the conversion of *p*-nitrophenyl phosphate to *p*-nitrophenol [28]. Optical density was determined by a microplate reader (Bio-Rad, Hercules, CA, USA) at a wavelength of 405 nm.

### Calcium contents

To evaluate late osteogenic differentiation, MC3T3-E1 cells were seeded at a concentration of 1 × 10^5^ cells/mL on a 24-well culture plate. The cells were incubated in C.M., O.M., and culture medium containing NDs. After 21 days of culture, the cells were washed with PBS, treated with 0.5 N HCl, and centrifuged at 13,500 rpm for 1 min. The resulting supernatant was used for calcium deposition measurement using a QuantiChrom Calcium Assay Kit (DICA-500, BioAssay Systems, Hayward, CA, USA), according to the manufacturer’s instructions. The amount of calcium produced was determined at 612 nm by a microplate reader.

### Gene expression

To evaluate the mRNA expression of the osteogenic differentiation markers (Runx-2, osteocalcin, and osteopontin), we performed a real-time polymerase chain reaction (RT-PCR). MC3T3-E1 cells (1 × 10^5^ cells/mL) were incubated in C.M., O.M., and culture medium containing NDs (100 μg/mL) and Alen-NDs (25, 50, and 100 μg/mL) in a 24-well culture plate. After 7 and 21 days of culture, cDNA was synthesized with 1 μg total RNA and oligo (dT) primer using the Superscript First-Strand Synthesis System (Bioneer Inc., Daejeon, Korea), according to the manufacturer’s instructions. The following oligonucleotide primers were used for RT-PCR: Runx-2, (F) 5′-ATG GCA TCA AAC AGC CTC TTC AGC A-3′, (R) 5′-CGT GGG TTC TGA GGC GGG ACA CC-3′; ALP, (F) 5′-GTG GAA GGA GGC AGA ATT GAC CA-3′, (R) 5′-AGG CCC ATT GCC ATA CAG GAT GG-3′; OCN, (F) 5′-TGA GAG CCC TCA CAC TCC TC 3′, (R) 5′-ACC TTTGCT GGA CTC TGC AC-3′; OPN, (F) 5′-GAG GGC TTG GTT GTC AGC-3′, (R) 5′-CAA TTC TCA TGG TAG TGA GTT TTC C-3′; GAPDH, (F) 5′-ACT TTG TCA AGC TCA TTT CC-3′, and (R) 5′-TGC AGC GAA CTT TAT TGA TG-3′. PCR amplification and detection were carried out on an ABI7300 Real-Time Thermal Cycler (Applied Biosystems, Foster, CA, USA) with the DyNAmo SYBR Green qPCR Kit (Finnzymes, Espoo, Finland). The relative mRNA expression levels of Runx-2, osteocalcin, and osteopontin were normalized to that of GAPDH. All results were confirmed by repeating the experiment three times.

### Statics

Quantitative data are presented as the mean ± standard deviation and comparisons were carried out using one-way ANOVA (Systat Software Inc., Chicago, IL, USA). Differences were considered statistically significant at 푃 < 0.05 (^∗∗^푃 < 0.01, ^∗∗∗^푃 < 0.001).

## Result

**Fig. 1:**
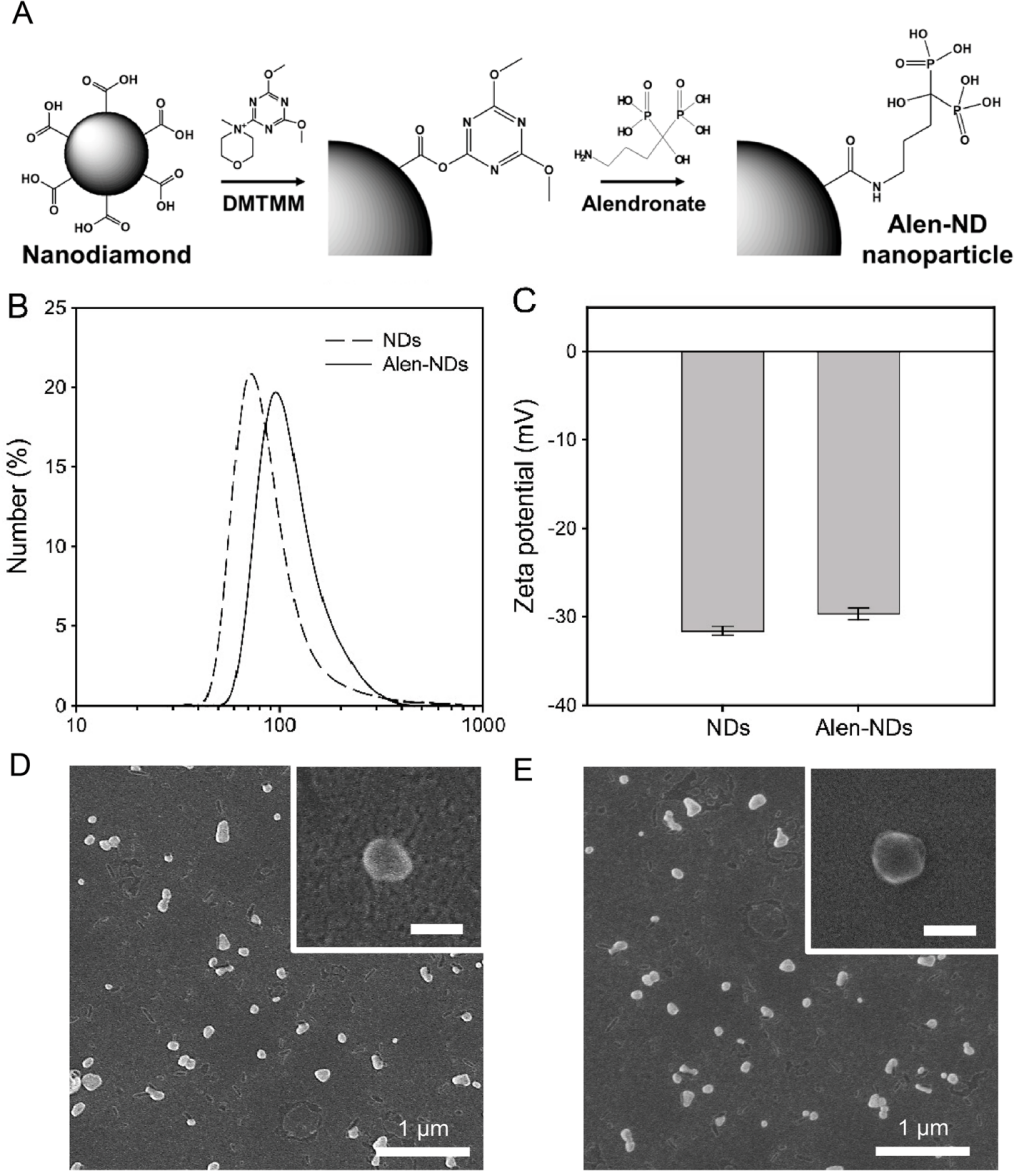
**A** Schematic illustration of the synthesis of Alen-NDs. **B** Size distribution, **C** zeta-potentials of NDs and Alen-NDs, and SEM images of (**D**) NDs and (**E**) Alen-NDs, where the inserts are magnified SEM images and scale bars represent 100 nm

Figure 1a shows the synthetic process for the conjugation of Alen onto NDs using DMTMM. Fig. 1b shows that the sizes of the NDs (94.5 ± 2.6 nm) and Alen-NDs (118.8 ± 4.5 nm). Zeta-potentials of NDs and Alen-NDs were − 31.7 ± 0.4 and − 29.7 ± 0.7 mV, respectively (Fig. 1c). Figure 1c and d show the SEM images of the NDs and Alen-NDs, respectively, indicating no significant change in morphology.

**Fig. 2:**
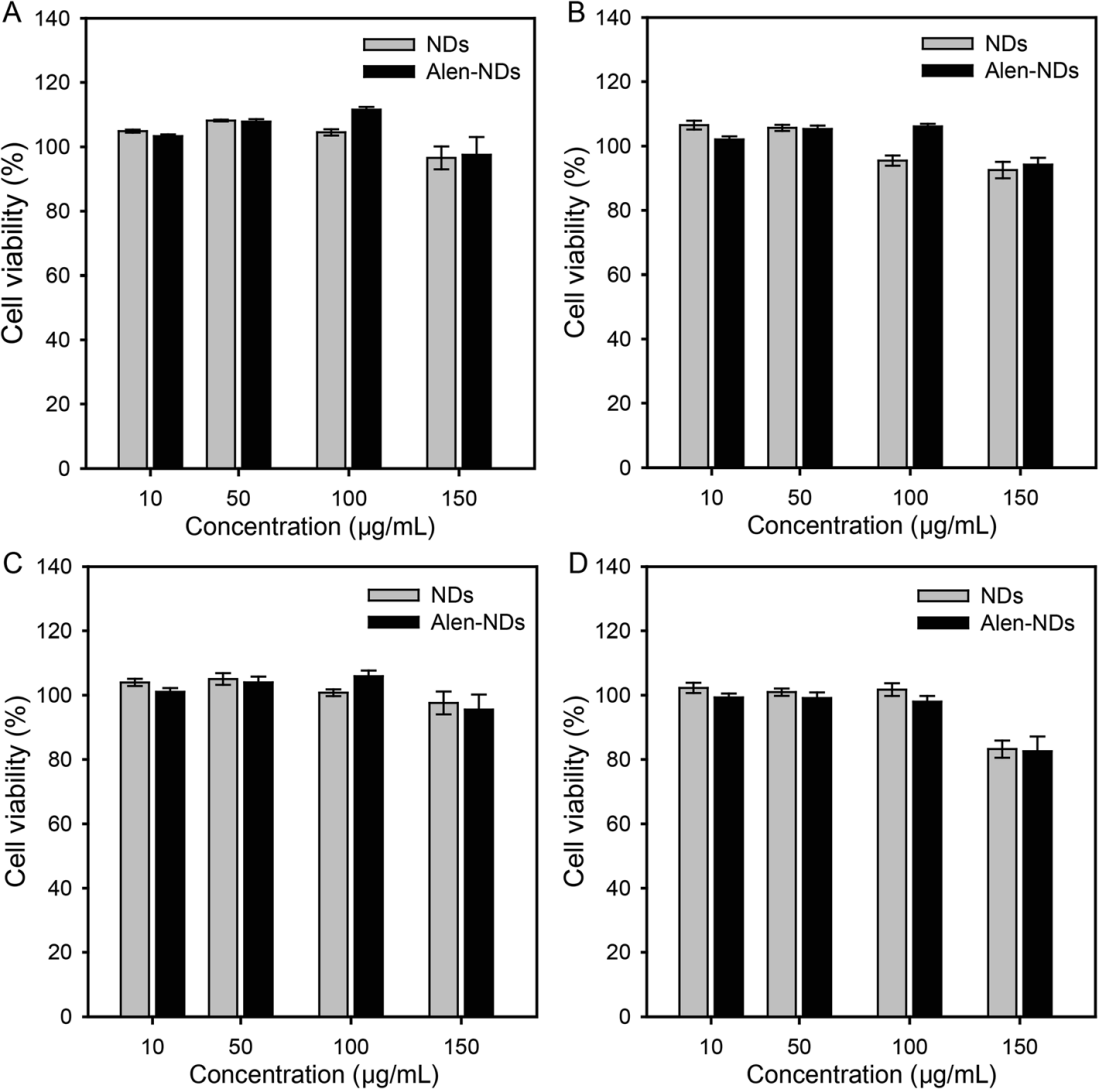
Viabilities of (**A**, **B**) NIH/3 T3 and (**C**, **D**) MC3T3-E1 cells after treatment with NDs and Alen-NDs for (**A**, **C**) 24 h and (**B**, **D**) 48 h

**Fig. 3:**
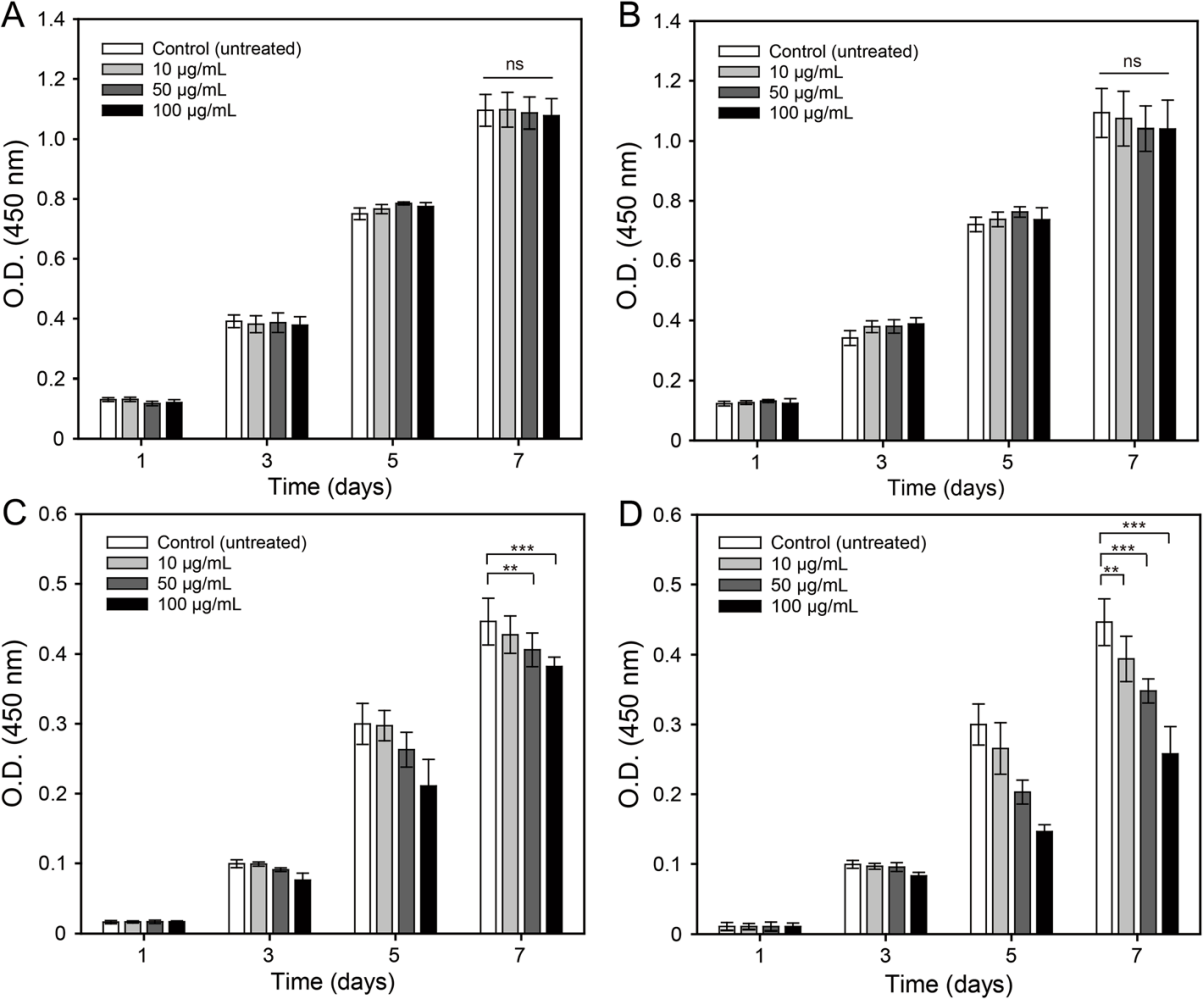
Proliferation of (**A**, **B**) NIH3T3 and (**C**, **D**) MC3TE-E1 cells treated with (**A**, **C**) NDs and (**B**, **D**) Alen-NDs (10, 50, and 100 μg/mL) after 1, 3, 5, and 7 days (ns: no significant difference, **p < 0.01, ***p < 0.001)

Figure 2 shows the viabilities of NIH/3 T3 and MC3T3-E1 cells for 24 and 48 h after treatment with NDs and Alen-NDs. There was no significant cytotoxicity in all samples at the concentration less than 100 μg/mL at 24 and 48 h. The viability of MC3T3-E1 was reduced to 82.5% at the concentration of 150 μg/mL at 48 h. Therefore, all the experiments were conducted at a concentration of less than 100 μg/mL. Figure 3 shows the cell proliferation of the NIH/3 T3 and MC3T3-E1 cells treated with NDs and Alen-NDs. Both the cells gradually proliferated over time. There were no significant differences in proliferation rates of NIH/3 T3 cells between NDs and Alen-NDs in all concentrations. However, the treatment of NDs and Alen-NDs significantly reduced the proliferation rate of MC3T3-E1 cells. The higher concentration of the Alen-ND resulted in the lower proliferation rate of the cells. At a concentration of 100 μg/mL, NDs reduced the proliferation rate of MC3T3-E1 cells by 14.4% (4.2 and 9.0% reduction at concentrations of 10 and 50 μg/mL, respectively) compared to the untreated group. In contrast, at a concentration of 100 μg/mL, Alen-NDs caused reduction in the proliferation rate by 42.2% at concentration of 100 μg/mL (11.7 and 22.0% reduction at concentration of 10 and 50 μg/mL) at 7 days.

**Fig. 4:**
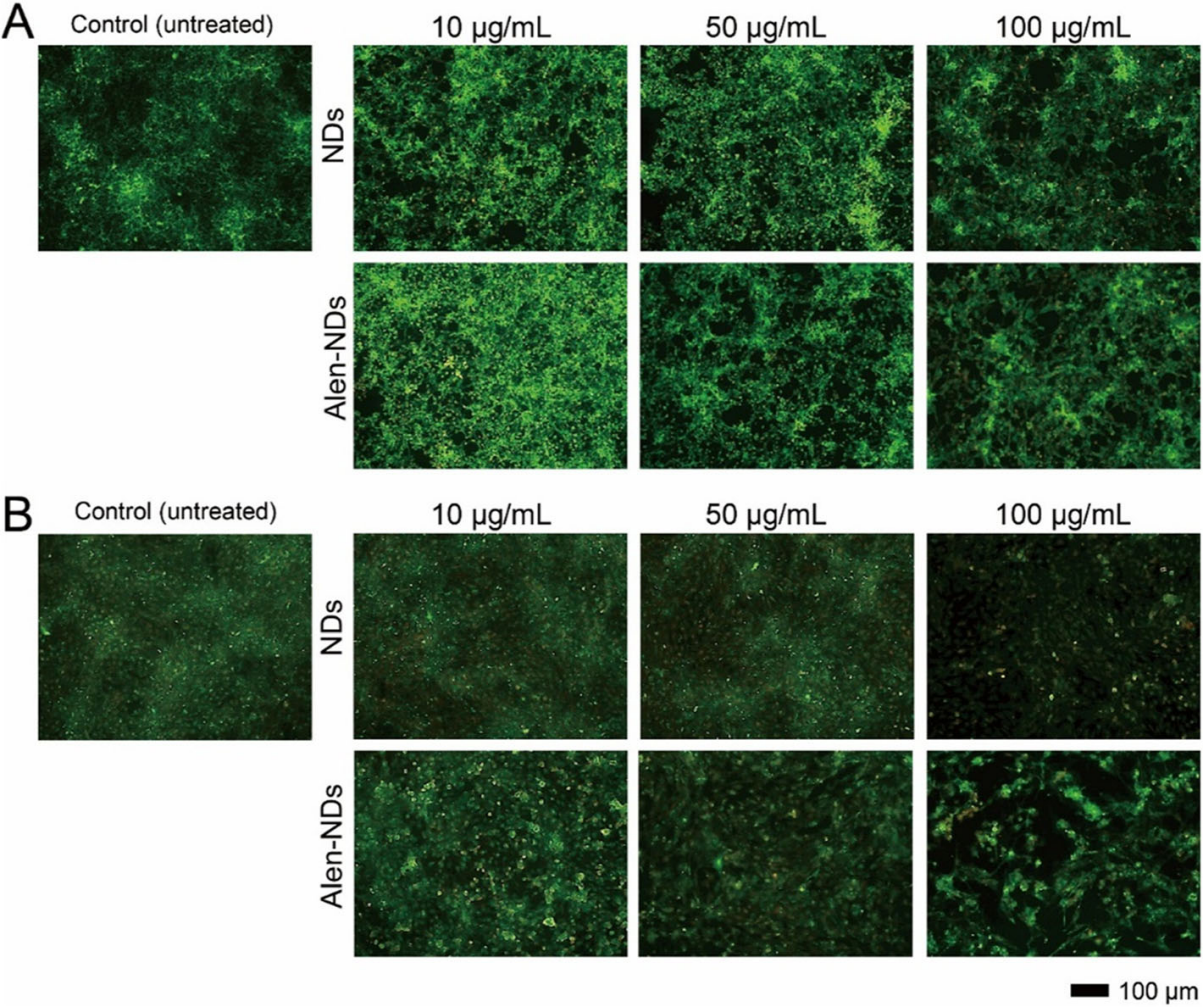
Fluorescence microscopy images of (**A**) NIH/3 T3, and (**B**) MC3T3-E1 cells, treated with NDs and Alen-NDs (10, 50, and 100 μg/mL) for 24 h, followed by LIVE/DEAD staining (merged images)

Live/Dead staining was conducted to verify the reason for the reduction in proliferation rate (observed in Fig. 3). Figure 4 shows the Live/Dead staining images of NIH3T3 and MC3T3-E1 cells treated with NDs and Alen-NDs according to concentrations (10, 50, and 100 μg/mL) at 7 days. There were few dead cells during culture with NDs and Alen-NDs, suggesting that most cells were still alive after treatment of NDs and Alen-NDs less than 100 μg/mL of concentration.

**Fig. 5:**
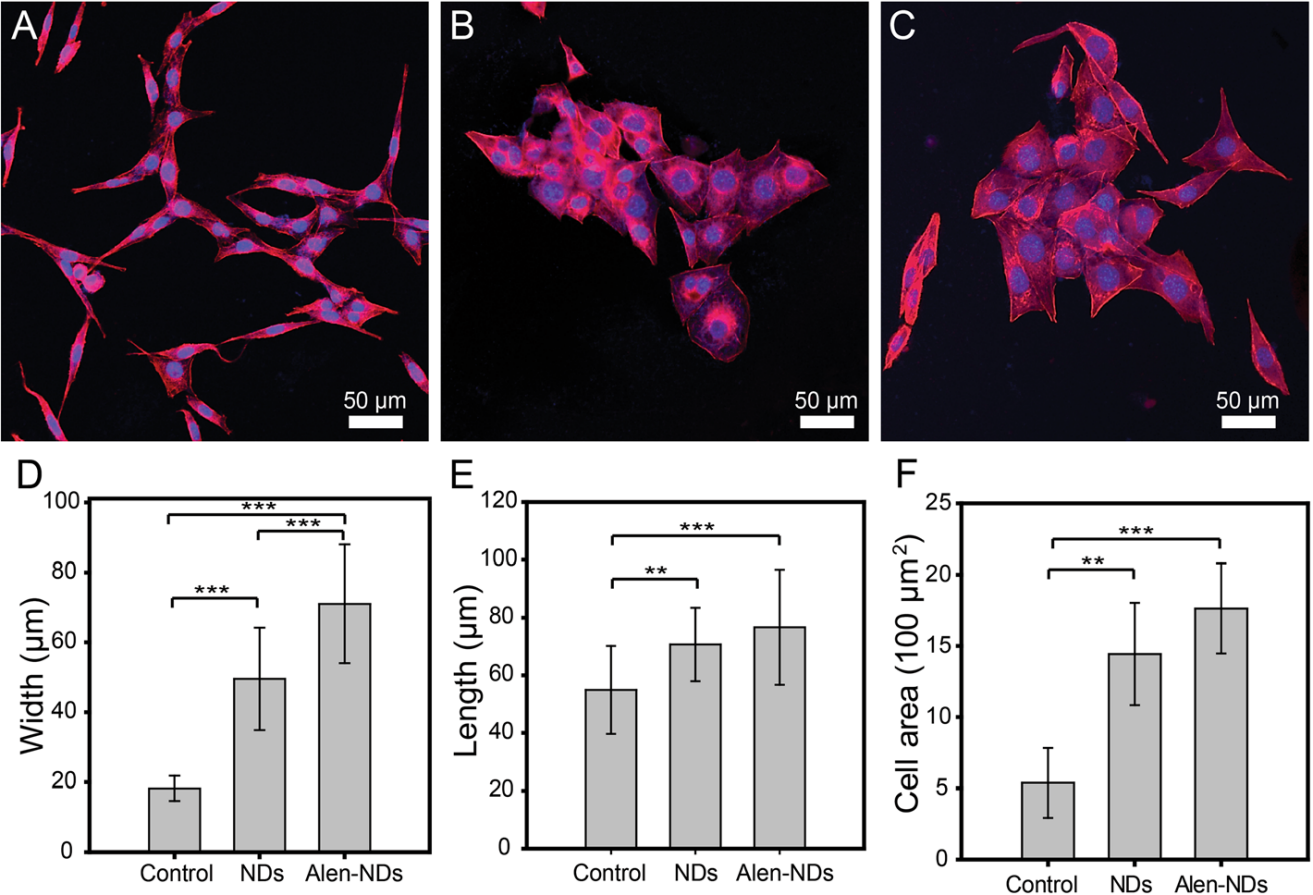
Confocal microscopy images of MC3T3-E1 cells treated with (**A**) only DMEM (control), (**B**) NDs, and (**C**) Alen-NDs (100 μg/mL) at 7 days. The (**D**) width, (**E**) length, and (**F**) cell area of the MC3T3-E1 cells treated with only DMEM (control), NDs, and Alen-NDs (100 μg/mL) at 7 days (**p < 0.01, ***p < 0.001)

Confocal microscopy was used to analyze the morphological change of MC3T3-E1 cells. Figure 5 shows confocal microscopy images of MC3T3-E1 cells treated by NDs and Alen-NDs, followed by dual staining. The MC3T3-E1 cells treated with NDs and Alen-NDs (100 μg/mL) showed more changes in cuboidal shapes from a fibroblastic shape, compared to the untreated group (control group). The width of MC3T3-E1 cells treated with NDs and Alen-NDs were 49.55 ± 14.68 and 71.00 ± 16.97 μm, respectively, having significantly higher width than the control group (18.25 ± 3.59 μm). Length and cell area of NDs and Alen-NDs also significantly increased, compared to the control group (Length: 54.93 ± 15.25 μm for control, 70.68 ± 12.72 μm for NDs, and 76.68 ± 19.97 μm for Alen-NDs. Cell area: 537.82 ± 244.88 μm^2^ for control, NDs: 1442.90 ± 359.94 μm^2^ for NDs, and 1763.06 ± 316.12 μm^2^ for Alen-NDs).

**Fig. 6:**
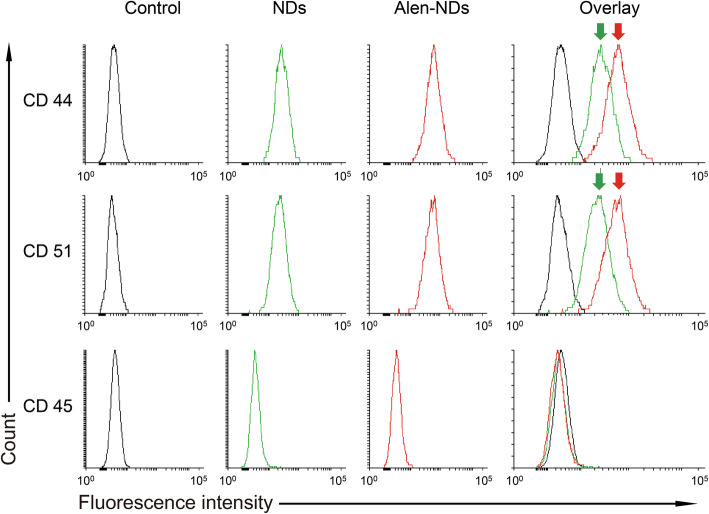
Flow cytometry analysis of MC3T3-E1 cells treated with (**A**) only DMEM (control), (**B**) NDs, and (**C**) Alen-NDs (100 μg/mL) at 7 days. Each group was treated with CD44, CD51 (positive control), and CD45 (negative control)

**Fig. 7:**
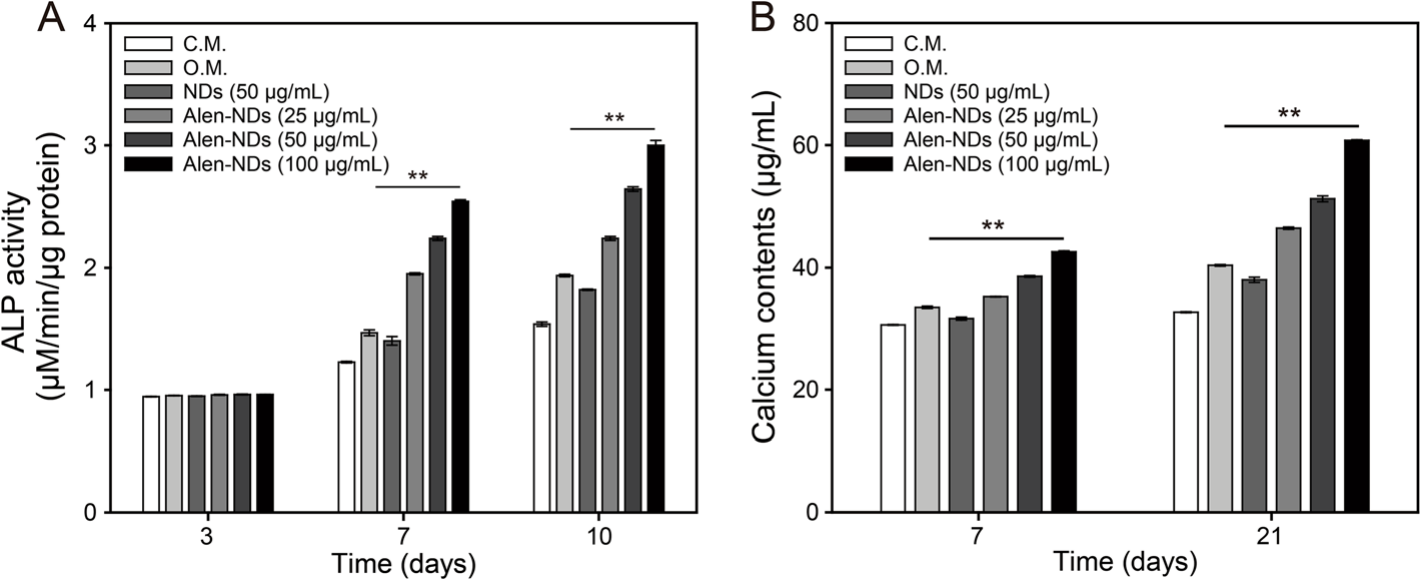
(**A**) ALP activity and (**B**) calcium contents of MC3T3-E1 cells treated with C.M., O.M., NDs (100 μg/mL), and Alen-NDs (25, 50, and 100 μg/mL) after 3, 7, and 10 days. A bar beneath the *symbol indicates statistical significance (**p < 0.01) across all 5 experimental groups at each time point

Figure 6 shows the expression of CD44, CD45, and CD51 of MC3T3-E1 cells treated with NDs and Alen-NDs at a concentration of 100 μg/mL, measured by flow cytometry. The MC3T3-E1 cells treated with NDs and Alen-NDs showed higher CD44 and CD51 expression levels than the untreated group. Besides morphological and biomarker analysis, Fig. 7a shows the ALP activity of MC3T3-E1 cells treated with NDs (100 μg/mL) and Alen-NDs (25, 50, and 100 μg/mL) after 3, 7, and 10 days, where culture medium (C.M.) and osteogenic medium (O.M.) were used as a negative and positive control, respectively. The ALP activity of MC3T3-E1 cells treated with NDs and Alen-NDs was gradually increased up to 10 days. The ALP activity of MC3T3-E1 cells treated with NDs is smaller than that of the O.M. treated group, indicating the relatively low differentiation effect of NDs. However, Alen-NDs showed a higher osteogenic differentiation than the O.M. group, even in a low concentration of 25 μg/mL. Alen-NDs at a concentration of 100 μg/mL showed a 1.55-fold higher ALP activity than that of the O.M. group at 10 days. Similar to the ALP activity results, the amounts of calcium contents treated with Alen-NDs were significantly higher than those treated with O.M. (Fig. 7b). The Alen-NDs at a concentration of 100 μg/mL showed 1.50-fold higher calcium contents than those of the O.M. group at 21 days.

**Fig. 8:**
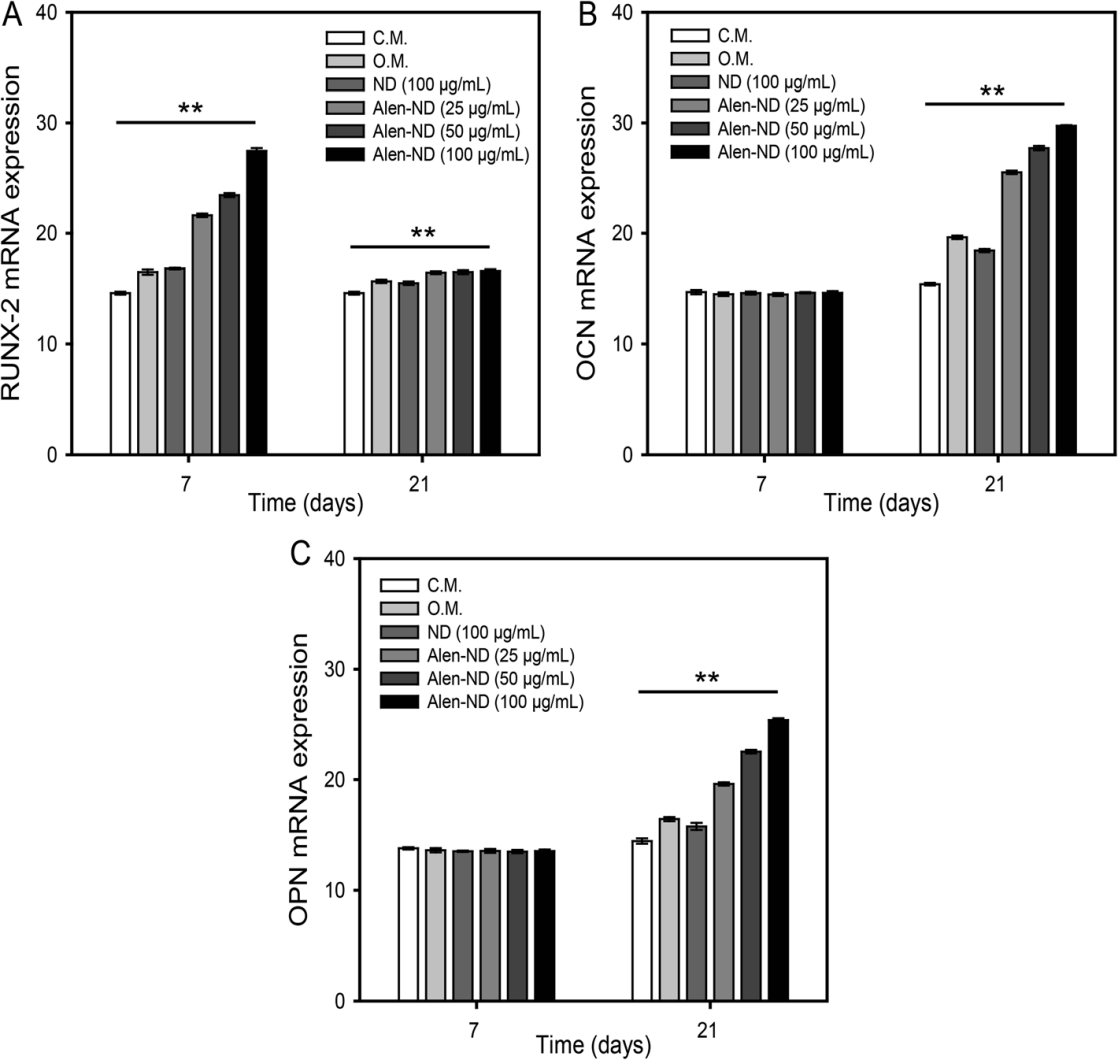
RT-PCR analysis for mRNA levels of (**A**) RunX-2, (**B**) osteocalcin (OCN), and (**C**) osteopontin (OPN) expression of MC3T3-E1 cells treated with C.M., O.M., NDs (100 μg/mL), and Alen-NDs (25, 50, and 100 μg/mL) after 7 and 21 days. A bar beneath the *symbol indicates statistical significance (**p < 0.01) across all 5 experimental groups

Figure 8 shows the mRNA expression for Runx-2, osteocalcin (OCN), and osteopontin (OPN) of MC3T3-E1 cells treated with NDs (100 μg/mL) and Alen-NDs (25, 50, and 100 μg/mL) after 7 and 21 days. The Runx-2 expression at 7 days showed a similar tendency to the ALP activity and calcium contents results. The MC3T3-E1 cells treated with Alen-NDs at a concentration of 100 μg/mL showed a 1.66-fold higher expression level than the group treated with O.M. (Fig. 8a). At 21 days, Runx-2 expression levels were all decreased. However, there were significant differences in each group at 21 days and Alen-NDs showed the highest expression level. Figure 8b and c show that neither the OCN nor OPN expression levels showed significant differences in each group at the relatively early stage (7 days). However, both the OCN and OPN expression levels were increased at 21 days. The levels of OCN and OPN expression in MC3T3-E1 cells treated with 100 μg/mL Alen-NDs were (1.51 and 1.54)-fold higher, respectively, than those of the O.M. group.

## Discussion

In this work, Alen-NDs were synthesized to confirm the osteogenic differentiation of MC3T3-E1 cells. Alen-NDs were synthesized by forming an amide bond using DMTMM as a coupling reagent. The DMTMM activated carboxyl groups of NDs and induced an amide coupling reaction with the Alen. DMTMM was used in this chemical synthesis, as it is non-toxic, and can be completely removed from the mixture after a reaction [29]. The increase in the size of Alen-NDs might be due to Alen-conjugation on the NDs surface. It has been reported that the nanoparticles of about 100 nm size showed high cellular uptake efficiency in in vitro tests [30]. Despite conjugation, there was no significant difference in zeta-potential between NDs and Alen-NDs, which is due to the presence of phosphate groups of Alen [31]. Both the NDs and Alen-NDs showed a high negative charge, indicating good long-term colloidal stability. The cell viabilities of the NDs and Alen-NDs were first evaluated to determine their concentrations for in vitro study. MC3T3-E1 (osteoblast precursor) cell line was chosen as a bone model cell, whereas NIH/3 T3 cells were used as control. The viabilities of NIH/3 T3 and MC3T3-E1 cells for 24 and 48 h after treatment with NDs and Alen-NDs showed no significant cytotoxicity in all samples at the concentration less than 100 μg/mL. This result suggested the non-cytotoxicity of NDs. The treatment of NDs and Alen-NDs significantly reduced the proliferation rate of MC3T3-E1 cells. The Alen-ND resulted in a lower proliferation rate of the cells than NDs. Many studies have reported that differentiation reduces the proliferation rate of osteoblast [32–34]. Therefore, this result may indicate differentiation of MC3T3-E1 cells induced by Alen-NDs. At Live/Dead staining images, there were few dead cells during culture with NDs and Alen-NDs, suggesting that reduced proliferation rate was not due to cell death. In confocal analysis, the enhanced cell area can be considered as evidence of osteoblast differentiation of MC3T3-E1 cells induced by the up-regulation of the cytoskeleton and focal adhesion [35]. In addition, Alen-NDs caused more cell morphological changes than NDs, indicating the synergistic effect of Alen and NDs. Biomarkers, such as CD44 and CD51, were used to confirm the effect of Alen-NDs on the differentiation of MC3T3-E1 cells. The CD44 and CD51 are expressed during osteoblast differentiation and can be used as biomarkers of bone formation [36]. CD45 is not an osteoblast differentiation biomarker and served as a negative control. Alen-NDs induced more expression of CD44 and CD51 than only NDs, indicating effective osteogenic differentiation. There was no significant difference among the samples in the CD45 expression levels. It has been reported that cellular stiffness improved osteogenic differentiation [37]. Therefore, we can assume that the ND-based nanoparticles taken up by cells increase the stiffness of MC3T3-E1 cells, resulting in the induction of osteogenic differentiation. Note that NDs can enhance the expression levels of CD44 and CD45. In addition, the expression level of both the biomarkers was more increased by the treatment of Alen-NDs. ALP activity is a biomarker commonly used to confirm early osteoblastic differentiation [38, 39]. The high ALP activity indicated a high osteogenic differentiation ability of Alen-NDs. These results confirm that Alen-NDs had a positive effect on the early stages of osteoblast differentiation. The calcium contents were evaluated up to 21 days since it is a late-stage differentiation marker of osteoblast [40, 41]. The mRNA expression for Runx-2, OCN, and OPN was used to confirm the effect of Alen-NDs on the differentiation of MC3T3-E1 cells. Runx-2 is an essential transcription factor for osteogenic differentiation and is widely used to confirm osteoblast differentiation [42, 43]. At 21 days, Runx-2 expression levels were all decreased, since Runx-2 induces the expression of bone matrix protein genes at an early stage of osteoblast differentiation [44]. OCN and OPN are late-stage markers of osteoblast differentiation [45, 46]. OCN is the main non-collagenous protein component of the bone extracellular matrix and OPN is an extracellular matrix cell adhesion protein, both of which are used as specific indicators of osteoblast differentiation [47]. OCN and OPN expression levels showed no significant differences in each group at the relatively early stage (7 days), because of their late-expression properties. Both the increased OCN and OPN expression levels at 21 days indicate a positive effect on the late stages of osteoblast differentiation. Taken together, these results demonstrate that Alen-NDs can effectively induce the osteogenic differentiation of MC3T3-E1 cells without cell death.

## Conclusions

In summary, we successfully demonstrated the superior performance of Alen-NDs for osteogenic differentiation in terms of cell proliferation, morphological changes, FACS analysis, ALP activity, calcium contents, and mRNA expression levels. The enhanced osteogenic differentiation is attributed to the synergistic effects of Alen and NDs. Although the osteogenic differentiation effect of only NDs was slightly lower than the osteogenic medium, these NDs alone exhibited a favorable osteogenic effect and also served as good carriers to effectively deliver and internalize drugs into cells. The mechanisms on the uptake and behavior of the Alen-NDs in cells are important for understanding the fate and therapeutic efficacy of the nanoparticles, because the cellular uptake, targeting, and intracellular behaviors of nanoparticles are greatly affected by their physicochemical properties such as size, shape and surface properties. As a next goal, we are planning to conduct experiments to assess the behaviors of the Alen-NDs after the cellular uptake. We believe that Alen-NDs have great potential to enhance osteogenic differentiation, ultimately increasing bone formation. Our next goal will focus on the in vivo evaluation of Alen-NDs as a therapeutic agent for osteoporosis treatment.

## Data Availability

For data requests, please contact the authors.
